# First principles study of electrocatalytic behavior of olivine phosphates with mixed alkali and mixed transition metal atoms†

**DOI:** 10.1039/d0ra02577a

**Published:** 2020-08-06

**Authors:** Arup Chakraborty, Sooraj Kunnikuruvan, David Zitoun, Dan T. Major

**Affiliations:** Department of Chemistry, Institute for Nanotechnology & Advanced Materials, Bar-Ilan University Ramat-Gan 52900 Israel majort@biu.ac.il +972 3 738 40 53 +972 3 531 73 92

## Abstract

Lithium transition metal olivine phosphates are well known Li-ion battery cathode materials, but these materials can also be used as electrocatalyst. Recent experimental studies showed that olivine phosphates with mixed alkali metals (Li and Na) and mixed transition metals (Ni and Fe) provide better electrocatalytic activity compared to single alkali and transition metal alternatives. In the current work, we analyzed the role of alkali metals, transition metals and vacancies on the reactivity of a series of olivine phosphates with different stoichiometries using first principles calculations. To this end, we investigated the adsorption of water at the surface of these materials. We found that water binds preferably at Ni surface sites for materials devoid of alkali ion vacancies. We further found correlation between the calculated adsorption energy with experimentally measured overpotentials for a series of olivine phosphates. Additionally, we found correlation between the adsorption energy of the systems with the total charge polarization of surface and adsorbate. To explain the computed trends, we analyzed the occupancies of the partial density of states of the Ni and Fe 3d states and Bader atomic charges.

## Abstract 2

Li transition metal olivine phosphates are proposed as good cathode materials and electrocatalysts for OER. This first principles study provides new insights into the catalytic performance of olivine phosphates with mixed alkali, mixed transition metal, and Li vacancies.

## Graphical abstract

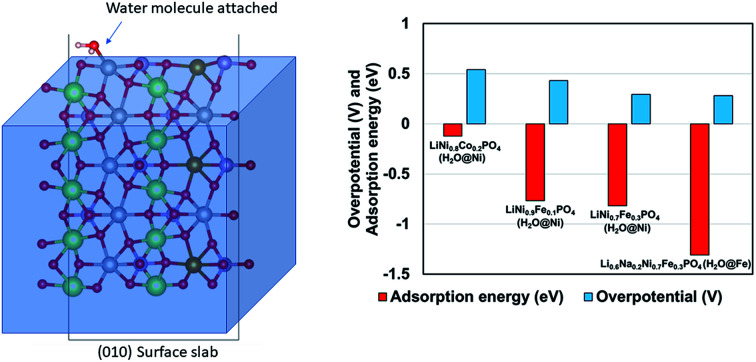

## 1 Introduction

Lithium transition-metal olivine phosphates with the general formula LiMPO_4_ (M = Fe, Co, Ni, Mn) are promising and environmentally benign energy storage materials applied as positive electrodes in Li-ion batteries. These materials are cost effective and provide good thermal stability during lithiation and delithiation.^1–5^ However, olivine phosphates with single transition-metal (TM) composition, such as LiFePO_4_, suffer from low electronic conductivity and poor redox kinetics.^6^ There are many recent studies on mixed transition metal olivine systems (
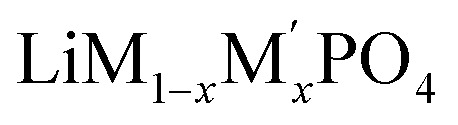
; M and M′ = Fe, Ni, Co, Mn) by various group which have shown ability to overcome these issues.^7–13^ Beyond this, the olivine phosphates are also known as good electrocatalyst for oxygen evolution reactions (OER).^14–17^ Excellent OER electrocatalysts, such as ruthenium, iridium and their oxides exist, but these are costly,^18–23^ and there is an ongoing search for non-noble metal catalysts for OER.^24–29^ Olivine phosphate materials can provide cost-effective alternatives to noble metal catalysts like Pt and Ir, and Fe doped Ni- and Co-based olivine phosphate were found to be promising OER active materials.^30,31^ Recently Gershinsky *et al.* synthesized mixed alkali (Li/Na) and mixed transition metal olivine phosphates and tested their OER activity.^32,33^ They showed that a particular combination of alkali metals (Li and Na) and transition metals with specific (1 M to 6 M) KOH concentration can be an excellent OER catalyst. Moreover, this study also indicated that defects in the olivine phosphate improved the OER catalytic activity.

Olivine phosphates belong to the olivine family which has an orthorhombic structure within the *Pnma* space group. In olivine structures, Li ions are arranged in edge sharing octahedra and transition metals (TMs) are in a corner sharing octahedral environment with oxygen atoms, whereas phosphorus ions are in a tetrahedral environment. The strong P–O covalent bonds provide the material with good thermodynamic stability, by reducing the release of oxygen.^6^ Morgan *et al.* showed that Li ions diffuse in one dimensional channels along the *b*-direction (*i.e.* the [010] direction in *Pnma* structures).^34^ Later Islam *et al.* employed computational modelling to show that the [010] direction is the favorable one for Li diffusion, while Nishimura *et al.* reached the same conclusion using an experimental approach.^35–37^ Further Wang *et al.* found that the (010) surface plane has lower surface energy compare to other planes, like (100) and (101).^38^

In the present work, we perform first principles-based computations to understand the effect of mixed alkali and transition metals and defects on the reactivity of olivine phosphates by looking at the adsorption of water on the surface of these materials. OER involves different intermediate steps where binding of H_2_O, OH, O, OOH occurs at the metal site of the surface of the electro-catalyst.^16,39^ It has been shown in earlier studies that these different steps are linearly correlated.^15,16,40–45^ Thus, the metal–oxygen interaction along the OER cascade is likely to play an important role in the rate limiting step.^40,45,46^ Although water binding is presumably not the rate limiting step for OER,^16,46^ water binding is expected to reflect on the ability of the olivines to bind oxygen. Here, we systematically explore the binding of a water molecule at the surfaces of mixed alkali and mixed transition metal olivine phosphate using density functional theory (DFT) calculations. Our studies mainly focus on Li_0.8_Na_0.2_Ni_0.7_Fe_0.3_PO_4_ without and with Li-vacancies. We further chose four different compounds LiNiPO_4_, LiNi_0.7_Fe_0.3_PO_4_, LiNi_0.8_Co_0.2_PO_4_, and LiNi_0.9_Fe_0.1_PO_4_, respectively for a comparative study following the work of Gershinsky *et al.*^33^ Based on these studies we qualitatively correlate between the observed electrochemical activity for OER and the probability of binding water on the surface of these materials with and without Li-vacancies. To the best of our knowledge, this is the first computational study focusing on the surface reactivity of olivine phosphate-based materials with application to OER.

## 2 Methods and models

### 2.1 Computational details

All the calculations were performed using plane wave-based DFT, as implemented in the Vienna *Ab initio* Simulation Package (VASP).^47,48^ We employ projector augmented wave (PAW) potentials,^49^ in conjunction with the Perdew–Burke–Ernzerhof (PBE) functional^50^ with Hubbard−*U* correction^51^ (Dudarev's method^52^). The applied effective value of *U* for Ni, Co, and Fe are 5.96, 5.7, and 4.3 eV, respectively.^7^ The cut-off energy value for the plane wave basis was set to 520 eV. The convergence limit for the energy in a self-consistent run was set to 10^−5^ eV, whereas 0.01 eV Å^−1^ was used for the force convergence per atoms during geometry optimization. We chose a *Γ*-centered *k*-mesh of 8 × 4 × 1 to sample the irreducible part of the Brillouin zone.

### 2.2 Structural model

**Fig. 1:**
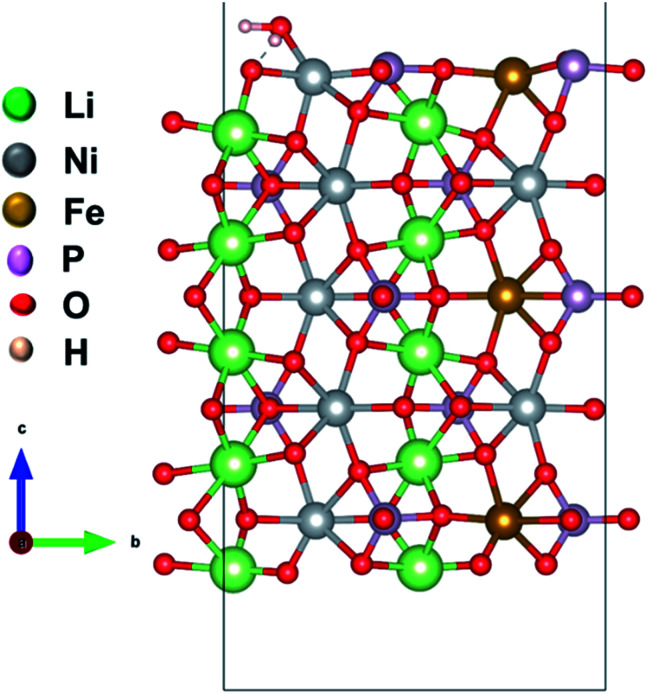
Model of a (010) surface slab with a water molecule adsorbed to Li_10_Ni_7_Fe_3_P_10_O_40_. Li, Ni, Fe, P, and O are in green, grey, brown, magenta and red color, respectively.

We considered four formula units to model a bulk orthorhombic structure of LiNiPO_4_ within the *Pnma* space group. To find the adsorption energy of water at the surface of the olivine phosphates, we generated a (010) plane surface slab, as this plane is the most stable.^35–37^ The slab model was created from an optimized bulk unit cell, and the slab model was reoptimized after generation. The lattice parameters for the surface slab model are *ca.* 4.8 Å and 10.2 Å. The thickness of the slab is approximately 13.7 Å, as this thickness produces converged values for calculated surface energies, as reported by Wang *et al.*^38^ A vacuum of 20 Å in the [010]-direction is employed to nullify the periodic image effect normal to the (010) plane. Our surface slab constitutes 10 formula units of LiMPO_4_. In this stoichiometric surface slab, one end is terminated with Li ions and other end is terminated with TMs. One water molecule is attached to a TM site as shown schematically in Fig. 1 and S1 in ESI.†

## 3 Results and discussion

In the following paragraphs we will first discuss bulk material properties, followed by a discussion of the electronic structure of the (010) surface and the adsorption of a single water molecule on this surface for different combinations of alkali and transition metals with and without defects.

Initially, we consider bulk orthorhombic LiNiPO_4_ within the *Pnma* space group. In this system, we find that the anti-ferromagnetic configuration is more stable than the ferromagnetic configuration by 28 meV (see Table S1 in ESI†). From electronic structure calculations, we observed that Ni is in a 2+ oxidation state and in a high spin state (calculated moments of Ni ions are *ca.* 1.8 *μ*_B_). In these pure systems we introduce one Na atom within the bulk unit cell consisting of four formula units. The formation energy (Δ*E*_f_) is calculated using the following formula for Na doping at a Li site:Δ*E*_f_ = *E*(Li_0.75_Na_0.25_MPO_4_) − *E*(LiMPO_4_) − *μ*(Na) + *μ*(Li)where *E*(Li_0.75_Na_0.25_MPO_4_) and *E*(LiMPO_4_) are the ground state energies of Li_0.75_Na_0.25_MPO_4_ and LiMPO_4_ respectively. M is a TM, like Ni. Based on these calculations, we find that Na preferably replaces Li in LiNiPO_4_ system (see Table S2 in ESI†). Further we model a bulk unit cell in the presence of Li-vacancies (25%) and find that Na replaces Li in Ni–Fe mixed TM material. Replacement of Li by Na is expected since both Li and Na ions are in a +1 oxidation state.

**Fig. 2:**
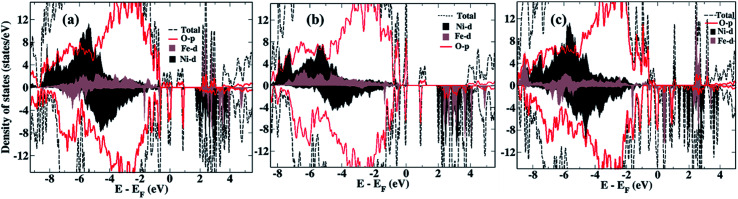
Density of states of the bare surfaces of (a) LiNi_0.7_Fe_0.3_PO_4_, (b) Li_0.8_Na_0.2_Ni_0.7_Fe_0.3_PO_4_, (c) Li_0.6_Na_0.2_Ni_0.7_Fe_0.3_PO_4_.

We now compare the density of states (DOS) of the bare surfaces of LiNi_0.7_Fe_0.3_PO_4_ (Fig. 2a) and Li_0.8_Na_0.2_Ni_0.7_Fe_0.3_PO_4_ (Fig. 2b). We observe that TM-3d states appearing near the Fermi energy mainly consist of Fe-3d orbitals, while the conduction band consists of Ni-3d states for both LiNi_0.7_Fe_0.3_PO_4_ and Li_0.8_Na_0.2_Ni_0.7_Fe_0.3_PO_4_ when there are no Li-vacancies. Hence, we expect that Ni ions will be reduced first during water adsorption rather than Fe, due to the presence of antibonding Ni-3d states. In presence of Li-vacancies (*e.g.* Li_0.6_Na_0.2_Ni_0.7_Fe_0.3_PO_4_), the scenario is different as both the occupied and unoccupied levels near the Fermi level are composed of Fe-3d states (Fig. 2c). Hence, water is expected to adsorb at Fe-sites in the case of Li-vacancies.

Further, we calculated the adsorption energy of water to understand the difference in reactivity of these surfaces. The formula for the adsorption energy (Δ*E*_ads_) isΔ*E*_ads_ = *E*(Li_*x*_Na_*y*_Ni_0.7_Fe_0.3_PO_4_·H_2_O) − *E*(Li_*x*_Na_*y*_Ni_0.7_Fe_0.3_PO_4_) − *E*(H_2_O)where *E*(Li_*x*_Na_*y*_Ni_0.7_Fe_0.3_PO_4_·H_2_O), *E*(Li_*x*_Na_*y*_Ni_0.7_Fe_0.3_PO_4_), *E*(H_2_O) are the energies of Li_*x*_Na_*y*_Ni_0.7_Fe_0.3_PO_4_ with a water molecule, bare surface of Li_*x*_Na_*y*_Ni_0.7_Fe_0.3_PO_4_ and a single water molecule, respectively. The (010) surface of LiNi_0.7_Fe_0.3_PO_4_ and Li_0.8_Na_0.2_Ni_0.7_Fe_0.3_PO_4_ with mixed transition metals, like Ni and Fe, contain two different metal atoms, Ni and Fe at the surface. Hence, the adsorption of a single water molecule can occur at any of Ni or Fe sites. The calculated adsorption energies at both TM sites are presented in Table 1.

**Table 1:** Adsorption energy (eV) for a single water molecule in Li_*x*_Na_*y*_Ni_0.7_Fe_0.3_PO_4_ at different TM sites

Systems	Δ*E*_ads_ (eV)	
	Water molecule attached at Ni site	Water molecule attached at Fe site
LiNi_0.7_Fe_0.3_PO_4_	−0.817	−0.372
Li_0.8_Na_0.2_Ni_0.7_Fe_0.3_PO_4_	−0.763	−0.299
Li_0.8_Ni_0.7_Fe_0.3_PO_4_	−0.680	−1.280
Li_0.6_Na_0.2_Ni_0.7_Fe_0.3_PO_4_	−0.743	−1.309

From the calculation of adsorption energy of a water molecule at different TM sites, we observe that the Ni site is preferable in the absence of Li vacancies (Table 1), in agreement with the expectation based on the computed DOS (*vide supra*). Moreover, both Ni and Fe are in a 2+ oxidation state, and the reduction potential of Ni^2+^ is higher than Fe^2+^, hence also supporting water adsorption at Ni sites. Unlike the above systems which are devoid of vacancies, the introduction of vacancies was found to change the favorable adsorption site from Ni to Fe as indicated by the adsorption energy calculations for Li_0.8_Ni_0.7_Fe_0.3_PO_4_ (Table 1). The preference for Fe sites can be ascribed to the presence of unoccupied states which consist of Fe 3d states closer to the Fermi level than the Ni 3d states, as discussed above.

In the absence of alkali ion vacancy, doping LiNi_0.7_Fe_0.3_PO_4_ with Na does not affect water adsorption to this surface significantly, as indicated by the comparable adsorption energy to that of Li_0.8_Na_0.2_Ni_0.7_Fe_0.3_PO_4_ (Table 1). However, the presence of alkali ion vacancy was found to increase the reactivity of the system significantly, as indicated by the reduction in adsorption energy of Li_0.8_Ni_0.7_Fe_0.3_PO_4_ by about 0.46 eV and the reduction in adsorption energy of Li_0.6_Na_0.2_Ni_0.7_Fe_0.3_PO_4_ by about 0.55 eV compared to LiNi_0.7_Fe_0.3_PO_4_ and Li_0.8_Na_0.2_Ni_0.7_Fe_0.3_PO_4_, respectively. Further a comparison of adsorption energies of Li_0.8_Ni_0.7_Fe_0.3_PO_4_ and Na-doped (Li_0.6_Na_0.2_Ni_0.7_Fe_0.3_PO_4_) indicates that Na doping have minor effect on water binding also in the presence of Li-vacancies. These results suggest that the increase in reactivity is largely due to the presence of vacancies and to a smaller extent Na-doping. We note that Li_0.8_Ni_0.7_Fe_0.3_PO_4_ was difficult to synthesize as indicated by Gershinsky *et al.*,^33^ whereas Li_0.6_Na_0.2_Ni_0.7_Fe_0.3_PO_4_ is reported in experiments.

We further calculated the difference in charge density after calculating charge density along the grid points for the bare surface, surfaces with water, and for the water molecule at the same position (in absence of slab) using the following formula:^53^Δ*ρ* = *ρ*(surface with water) − *ρ*(bare surface) − *ρ*(water)Here *ρ*(surface with water), *ρ*(bare surface), and *ρ*(water) are the charge densities for the respective systems. Then we estimated the charge polarization between systems by multiplying Δ*ρ* with the volume of the unit cell and with electronic charge, as shown in Table 2.

**Table 2:** Total charge polarization for different systems

System	Total charge polarization (electron charge/cell volume)	Adsorption energy (Δ*E*_ads_) (eV)
Li_0.8_Na_0.2_Ni_0.7_Fe_0.3_PO_4_	1.85 × 10^−4^	−0.763
LiNi_0.7_Fe_0.3_PO_4_	1.95 × 10^−4^	−0.817
Li_0.8_Ni_0.7_Fe_0.3_PO_4_	6.93 × 10^−4^	−1.280
Li_0.6_Na_0.2_Ni_0.7_Fe_0.3_PO_4_	7.00 × 10^−4^	−1.309

We observed that the adsorption energy increases (absolute values) with increasing charge polarization upon water binding to the surface slab. The charge polarization is more prominent in the presence of Li-vacancies than the doping of Na. Hence, these results also reveal that the reactivity is increased due to the presence of vacancies rather than Na-doping and this is accompanied by greater charge polarization.

We further calculated Bader charges at the atom sites (see Tables S3 and S4 in ESI†) following the method proposed by the Henkelman group.^54,55^ Based on this electronic structure analysis we provide a rationale for the lower adsorption energy of Li_0.6_Na_0.2_Ni_0.7_Fe_0.3_PO_4_. The Ni and Fe atoms at the top of the surface slab have lower Bader charges compared to atoms within the bulk of the slab due to the presence of dangling bonds at the surface. The same is true for P atoms and O atoms at the surface which have two neighboring metal atoms. There are substantial changes in the Bader charge of Fe atoms at the top of the surface and at the subsurface near the bottom layer in our model. Introduction of Na and presence of Li-vacancies affect the Bader charge as well and this is also observed from the change in the moment of Fe from *ca.* 3.8 *μ*_B_ to 4.2 *μ*_B_. This can be attributed to the change in bond length of the atoms to adopt the bigger sized Na atoms. Next, we observed that Ni and Fe atoms in the top layer have higher Bader charge values for Li_0.6_Na_0.2_Ni_0.7_Fe_0.3_PO_4_ (see Table S3 in ESI†) compared to Li_0.8_Ni_0.7_Fe_0.3_PO_4_. This can be the reason for the better binding of water and lower adsorption energy in Li_0.6_Na_0.2_Ni_0.7_Fe_0.3_PO_4_ compared to Li_0.8_Ni_0.7_Fe_0.3_PO_4_.

**Fig. 3:**
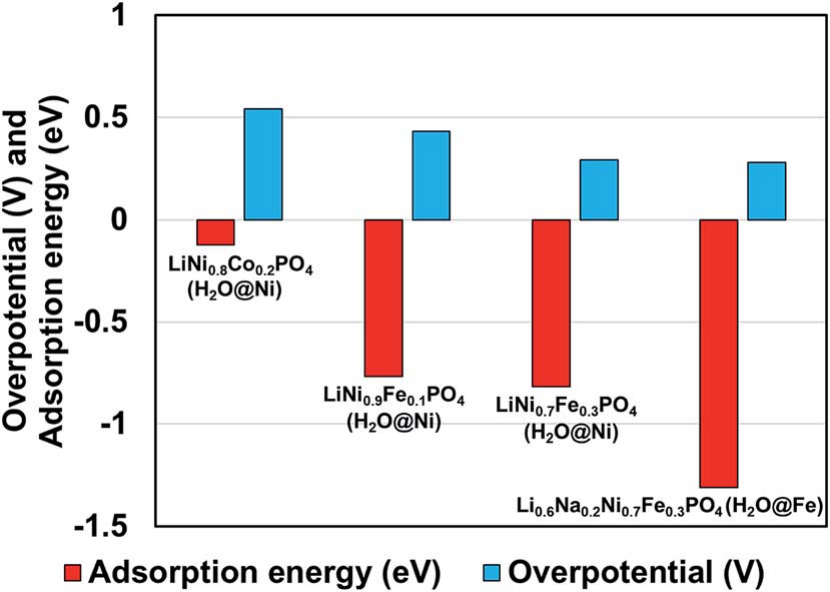
Comparison of calculated adsorption energy and experimentally^14,33^ measured overpotential for the olivine phosphates with mixed alkali and TM atoms.

We observed that for different combinations of alkali Li/Na and transition metals Ni/Fe or Ni/Co the calculated adsorption energy of a single water molecule at the (010) surface of the olivine phosphate follows a trend similar to that observed by Gershinsky *et al.*^33^ for the electrochemical activity of these systems for OER reactions based on overpotential measurements. We found that decreasing adsorption energy follows a trend of decreasing overpotential for the series of olivine phosphate, as shown in Fig. 3 and Table S5 (in ESI).†

## 4 Conclusions

We studied the binding of water at the surface of olivine phosphates with mixed alkali metals (Li and Na) and mixed TM atoms (Ni and Fe) using DFT. We considered the (010) plane of the olivine phosphate in our surface slab model, where one end is terminated with Li metal atoms and other end is terminated with TM atoms. The computed adsorption energy revealed that water preferably attaches to Ni sites in the absence of alkali ion vacancies. We explained this preference from the DOS of the bare surface where Fe-3d states compose the valence band whereas unoccupied Ni-3d states make up the conduction band, facilitating water binding *via* Ni sites. This trend of water adsorption changes to Fe-sites when Li-vacancies are present in the system. This is consistent with the DOS of the bare surface with Li-vacancies, which showed that Fe-3d states form the conduction band. This is also in line with the enhancement in calculated Bader charge at Fe sites in the presence of vacancies. Further we showed that there is qualitative correlation between the calculated adsorption energy of water at the surface and experimentally measured overpotential for the different combinations of mixed alkali and TMs olivine phosphates. Among the various experimentally reported systems considered in this study, Li_0.6_Na_0.2_Ni_0.7_Fe_0.3_PO_4_ with mixed alkali Li and Na in presence of Li-vacancies shows best electrocatalytic behavior experimentally and also is the most potent water binder. In short, this study provides new insights into the role of transition metals, Na-doping, and the effect of vacancy on the surface reactivity of a series of olivine phosphates with different stoichiometry. Importantly, the current calculations provide insights that may guide future experimental efforts.

## Conflicts of interest

There are no conflicts of interest to declare.

## Supplementary Material

RA-010-D0RA02577A-s001
