# Supplementary material for: First principles study of electrocatalytic behavior of olivine phosphates with mixed alkali and mixed transition metal atoms
Source: RSC Adv. 2020 Aug 6;10(49):29175–80. doi: 10.1039/d0ra02577a (PMC9055913; doi:10.1039/d0ra02577a)
Supplement: RA-010-D0RA02577A-s001 [file RA-010-D0RA02577A-s001.pdf]

Electronic Supplementary Material (ESI) for RSC Advances.
This journal is © The Royal Society of Chemistry 2020

Electronic Supplementary Information

First Principle Study of Electrocatalytic Behavior of Olivine Phosphates with Mixed Alkali and Mixed Transition Metal Atoms

Arup Chakraborty,[1] Sooraj Kunnikuruvan,[1] David Zitoun,[1] Dan T. Major[1,*]

[1] *Department of Chemistry and Institute for Nanotechnology & Advanced Materials, Bar-Ilan University, Ramat-Gan 52900, Israel*

Table S1 Energy difference between ferromagnetic (FM) and antiferromagnetic (AFM) configurations of Ni atoms in bulk $LiNiPO_4$.

Configuration	FM	AFM1 (C-type)	AFM2 (A-type)	AFM3 (G-type)
Relative energy (eV)	0.000	-0.028	-0.006	-0.021

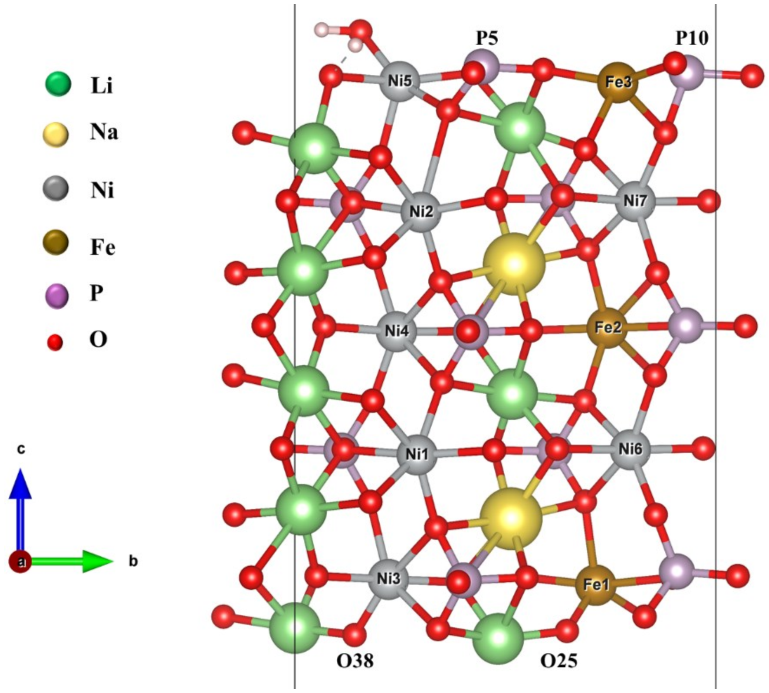

Figure S1 (010) surface slab model for $Li_{0.8}Na_{0.2}Ni_{0.7}Fe_{0.3}PO_4$ with a water molecule attached at Ni-site. Slab is confined along *c*-direction.

Table S2 Relative formation energy (eV) of Na doping at Li and Ni sites of $LiNiPO_4$.

Na at Li site ($Li_{0.75}Na_{0.25}NiPO_4$)	Na at Ni site ($LiNa_{0.25}Ni_{0.75}PO_4$)
0.000	1.039

Table S3 Bader charges for selected atoms in $Li_{0.8}Na_{0.2}Ni_{0.7}Fe_{0.3}PO_4$ without and with Li-vacancies. Atom numbers in this table are the same as in Figure S1.

Atomic site	Bare surface of $Li_{0.8}Na_{0.2}Ni_{0.7}Fe_{0.3}PO_4$	Bare surface of $Li_{0.6}Na_{0.2}Ni_{0.7}Fe_{0.3}PO_4$	Water attached to the Ni-site of $Li_{0.8}Na_{0.2}Ni_{0.7}Fe_{0.3}PO_4$	Water attached to the Ni-site of $Li_{0.6}Na_{0.2}Ni_{0.7}Fe_{0.3}PO_4$	Water attached to the Fe-site of $Li_{0.8}Na_{0.2}Ni_{0.7}Fe_{0.3}PO_4$	Water attached to the Fe-site of $Li_{0.6}Na_{0.2}Ni_{0.7}Fe_{0.3}PO_4$
Fe3 (at the top)	1.375	1.361	1.339	1.384	1.412	1.440
Fe1	1.715	1.690	1.724	1.703	1.716	1.733
Ni5 (at the top)	1.203	1.306	1.291	1.315	1.188	1.276
Ni3	1.296	1.282	1.290	1.291	1.295	1.293
Fe2	1.427	1.834	1.427	1.832	1.424	1.421
P5	2.349	3.617	2.770	3.596	2.330	2.921
P10	2.761	2.481	2.359	2.440	2.715	3.640
O25	0.867	0.903	0.867	0.909	0.685	0.853
O38	0.620	0.702	0.645	0.664	0.627	0.624

Table S4 Bader charges for selected atoms in $LiNi_{0.7}Fe_{0.3}PO_4$ without and with Li-vacancies. Atom numbers in this table are the same as in Figure S1.

Atomic site	Bare surface of $LiNi_{0.7}Fe_{0.3}PO_4$	Bare surface of $Li_{0.8}Ni_{0.7}Fe_{0.3}PO_4$	Water attached to the Ni-site of $LiNi_{0.7}Fe_{0.3}PO_4$	Water attached to the Ni-site of $Li_{0.8}Ni_{0.7}Fe_{0.3}PO_4$	Water attached to the Fe-site of $LiNi_{0.7}Fe_{0.3}PO_4$	Water attached to the Fe-site of $Li_{0.8}Ni_{0.7}Fe_{0.3}PO_4$
Fe3 (at the top)	1.571	1.356	1.333	1.346	1.406	1.439
Fe1	1.390	1.682	1.705	1.689	1.709	1.598

Ni5 (at the top)	1.164	1.315	1.284	1.317	1.170	1.207
Ni3	1.292	1.29	1.292	1.29	1.291	1.285
Fe2	1.429	1.829	1.427	1.832	1.427	1.837
P5	2.440	3.628	2.764	3.605	2.349	2.867
P10	2.765	2.511	2.342	2.459	2.761	2.886
O25	0.725	0.907	0.832	0.885	0.817	0.790
O38	0.653	0.665	0.653	0.656	0.657	0.708

Table S5. Comparison of adsorption energy and overpotential for the olivine phosphates with mixed alkali and TM atoms.

Theoretical composition	Experimental composition	Calculated ΔE_{ads} (eV)	Overpotential (V)
$LiNi_{0.8}Co_{0.2}PO_4$	$LiNi_{0.8}Co_{0.2}PO_4$	-0.123	0.54 (1 M KOH) [Ref.[1]]
$LiNi_{0.9}Fe_{0.1}PO_4$	$LiNi_{0.9}Fe_{0.1}PO_4$	-0.767	0.43 (1 M KOH) [Ref.[1]]
$LiNi_{0.7}Fe_{0.3}PO_4$	$LiNi_{0.75}Fe_{0.25}PO_4$	- 0.817	0.295 (1 M KOH) [Ref.[2]]
$Li_{0.6}Na_{0.2}Ni_{0.7}Fe_{0.3}PO_4$	$Li_{0.6}Na_{0.2}Ni_{0.7}Fe_{0.1}PO_4$	-1.309	0.280 (6 M KOH) [Ref.[1]]

References

1. Y. Gershinsky, M. Zysler, V. Shokhen, Y. Stone and D. Zitoun, *ACS Catal.*, 2019, **9**, 8355-8363.
2. S. Ma, Q. Zhu, L. Chen, W. Wang and D. Chen, *J. Mater. Chem. A*, 2016, **4**, 8149-8154.
